# Bridging bone and muscle: ontology-driven prioritization of candidate genes at the osteoporosis–sarcopenia interface by gene ontology reverse lookup

**DOI:** 10.3389/fgene.2026.1760184

**Published:** 2026-09-25

**Authors:** Simon Žetko, Lan Vukolić, Aljoša Škorjanc, Vladimir Smrkolj, Nejc Umek

**Affiliations:** Institute of Anatomy, Faculty of Medicine, University of Ljubljana, Ljubljana, Slovenia

**Keywords:** candidate gene prioritization, cytosolic DNA-sensing, GOReverseLookup, muscle-bone crosstalk, necroptosis, NF-κB signaling, osteosarcopenia

## Abstract

**Introduction:**

Osteosarcopenia reflects the co-occurrence of osteoporosis and sarcopenia, but the molecular processes shared between impaired bone and muscle homeostasis remain incompletely characterized. This study applied a transparent, hypothesis-generating annotation-based framework to prioritize candidate genes at the osteoporosis–sarcopenia interface.

**Methods:**

GOReverseLookup was applied to manually curated Gene Ontology term sets representing biological mechanisms relevant to osteoporosis and sarcopenia. A curated set of 367 GO terms representing key disease mechanisms served as input. Using Fisher’s exact testing with Benjamini–Hochberg correction and predefined ortholog-propagation settings incorporating *H. sapiens*, *M. musculus*, *R. norvegicus*, *D. rerio*, and *X. tropicalis* annotations, the pipeline prioritized genes meeting the significance criterion for both curated states of interest. Prioritized candidates were subsequently contextualized using targeted literature assessment, DisGeNET disease-association scores, Expression Atlas tissue-expression data and KEGG pathway analysis.

**Results:**

The primary analysis identified 37 candidate genes meeting the predefined significance criterion for both osteoporosis- and sarcopenia-related states of interest. The analysis highlighted a subset of less-characterized candidates, including *RBCK1*, *LGALS9*, *ZBTB7A* and *RPS3*, with detectable expression in the selected bone- and muscle-related tissue datasets. Recovery of previously described mediators of bone and muscle biology, including *TNFSF11* and *STAT3*, supported the biological plausibility of the prioritization framework. Pathway characterization predominantly indicated inflammatory and innate immune signalling, regulated cell death and osteoclast-related processes.

**Conclusion:**

GOReverseLookup generated a transparent, annotation-based candidate list for investigating shared osteoporosis- and sarcopenia-related mechanisms. These findings should be interpreted as hypothesis-generating prioritization results requiring independent experimental and clinical validation.

## 1 Introduction

Osteosarcopenia is an emerging geriatric syndrome and an increasingly recognized global health concern. It is defined as the co-occurrence of osteoporosis or low bone mass and sarcopenia; however, reported prevalence and case identification vary according to the diagnostic criteria used for the bone and muscle components of the syndrome. A recent meta-analysis estimated its pooled prevalence at approximately 21% (Huang et al., 2023). Osteoporosis is characterized by reduced bone mass and microarchitectural deterioration, resulting in skeletal fragility and a heightened susceptibility to fractures (Compston et al., 2019). Sarcopenia, by contrast, involves the progressive loss of skeletal muscle mass and strength, which underlies physical disability, diminished quality of life, and increased mortality. Although traditionally studied as distinct entities, accumulating evidence indicates substantial overlap in their pathogenesis and risk factors including aging, hormonal decline, nutritional deficiency and sedentary behavior (Clynes et al., 2021; Huang et al., 2023). The concurrent manifestation of both conditions synergistically exacerbates the risk of falls, fractures, and long-term disability (Clynes et al., 2021). In the present study, osteosarcopenia was not investigated in a clinically phenotyped patient cohort; rather, it was used as a conceptual framework for examining biological processes shared between osteoporosis and sarcopenia.

At the molecular level, osteoporosis and sarcopenia arise from distinct yet parallel disruptions of tissue homeostasis. In osteoporosis, bone resorption exceeds bone formation, leading to a progressive decline in bone mass and an increased risk of fracture (Huang et al., 2023). The balance between osteoclast-mediated resorption and osteoblast-driven formation is governed by several key signalling axes, including the receptor activator of nuclear factor κB ligand/receptor activator of nuclear factor κB/osteoprotegerin (RANKL/RANK/OPG) system, Wnt/β-catenin, and transforming growth factor-β/bone morphogenetic protein (TGF-β/BMP) pathways (Noh et al., 2020). Additional factors, such as estrogen deficiency in postmenopausal women and chronic inflammation, further exacerbate the imbalance in bone remodeling, contributing to a loss in bone density (Chen et al., 2021). In parallel, sarcopenia is driven by impaired muscle protein synthesis and enhanced proteolytic activity. Age-related changes in muscle architecture, including the selective atrophy of type II fibers and reduced satellite cell regenerative capacity, further compromise muscle maintenance and repair (Cho et al., 2022).

The pathological overlap between osteoporosis and sarcopenia arises from the interdependent signaling networks that link bone and muscle homeostasis. Both tissues originate from mesenchymal stem cells and remain closely interlinked through paracrine, endocrine and mechanical stimuli (Fagundes Belchior et al., 2020; Clynes et al., 2021). Myokines, such as myostatin, insulin-like growth factor 1 (IGF-1), irisin, and fibroblast growth factor 2 (FGF-2) have been implicated in bone remodeling (Michaud et al., 2013), whereas osteokines including osteocalcin and TGF-β influence muscle metabolism and contractile function (Zhao et al., 2024). The Wnt signalling pathway, essential for bone formation, also plays a pivotal role in muscle regeneration (Polesskaya et al., 2003). Chronic low-grade inflammation contributes to the atrophy of both tissues, as pro-inflammatory signals accelerate osteoclastogenesis and proteolysis (Michaud et al., 2013). Oxidative stress and hormonal dysregulation further amplify these pathological processes (Manolagas, 2010). The reciprocal communication between muscle and bone appears essential for maintaining musculoskeletal function. Disruptions in this signaling can contribute to osteosarcopenia. These insights underscore the need for therapeutic strategies that target skeletal fragility and muscle wasting simultaneously rather than in isolation (Sheng et al., 2023).

Identifying candidate genes involved in biological processes shared by osteoporosis and sarcopenia may support the development of mechanistic hypotheses and guide subsequent validation studies. Existing computational strategies address this objective from complementary perspectives. Genome-wide association studies (GWAS) and transcriptome-wide association studies (TWAS) can connect genetic variation with complex traits and genetically regulated expression, but interpretation of small-effect signals and causal genes remains challenging (Manolio et al., 2009; Wainberg et al., 2019). Phenome-wide association studies (PheWAS) invert the GWAS logic by scanning across clinical phenotypes, yet remain susceptible to phenotype misclassification and a substantial multiple-testing burden (Denny et al., 2016). Convergent functional genomics (CFG) and related evidence integration frameworks aim to prioritize loci by aggregating heterogeneous data, but their outputs can be dominated by well-studied pathways and literature bias (Niculescu and Le-Niculescu, 2010). Recent representation-learning approaches including hypergraph embeddings, protein-interaction graph neural networks and ontology vectorizations such as Onto2Vec and SmuDGE, have captured latent structure across Gene Ontology (GO), OMIM and HPO to predict disease genes. However, their application to the present study is constrained by two limitations: their predictions are less biologically auditable because they arise from latent-space proximity rather than traceable mechanisms (Alshahrani and Hoehndorf, 2018; Smaili et al., 2018), and their performance depends on existing disease-gene annotations, which remain sparse and biased for emerging composite phenotypes such as osteosarcopenia. Recent integrated omics studies have investigated osteoporosis–sarcopenia overlap using patient-derived bone and muscle transcriptomic and proteomic data (Chen et al., 2024), while causal-inference studies have applied bidirectional or multi-omics Mendelian randomization, in some cases combined with single-cell analysis, to evaluate relationships and potential therapeutic targets relevant to osteosarcopenia (Ma et al., 2022; Yang et al., 2025). These approaches are valuable for data-driven discovery and causal inference, but they require suitable patient-derived datasets or disease-specific annotations that remain limited for osteosarcopenia. GOReverseLookup addresses a different methodological need: transparent candidate prioritization for early-stage hypothesis generation when the phenotype is biologically complex, incompletely annotated, and not yet supported by large dedicated multi-tissue datasets. Instead of starting from genetic variants or patient-derived molecular profiles, researchers curate GO terms that define a phenotype or broader state of interest and the tool then evaluates which genes are statistically over- or under-represented in that user-defined state (Škorjanc et al., 2025). The aim of this study was to prioritize candidate genes enriched for GO annotations related to biological processes relevant to both osteoporosis and sarcopenia, and to characterize their biological plausibility using complementary literature-, database-, expression- and pathway-based resources.

## 2 Methods

Candidate genes were identified using GOReverseLookup, a computational tool that prioritizes genes based on their statistical association with a curated set of GO terms that collectively define a biological phenotype (Figure 1). The tool’s workflow consists of three main stages: input curation, annotation retrieval and statistical analysis. The only stage performed by the researcher is input curation, whereas all subsequent steps are handled automatically by GOReverseLookup.

**FIGURE 1:**
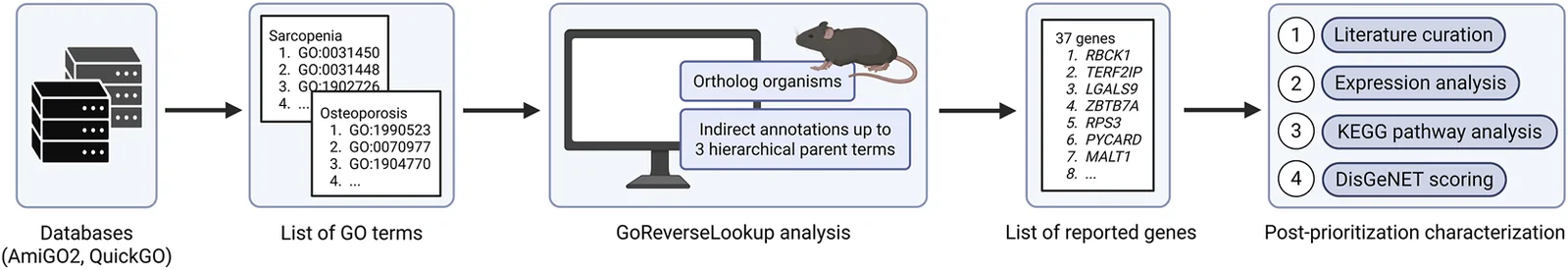
Methods overview. Targeted searches of Gene Ontology exploration and annotation resources AmiGO2 and QuickGO curated two GO-term sets representing biological mechanisms relevant to predefined states of interest (SOIs): osteoporosis and sarcopenia. GOReverseLookup analyzed term sets using the predefined research-model settings, including ortholog-derived annotations, indirect annotations propagated up to three hierarchical parent terms, and selected evidence-code filters. The analysis generated a prioritized set of 37 candidate genes enriched for GO annotations related to both osteoporosis- and sarcopenia-relevant SOIs. Prioritized candidates were subsequently characterized using targeted literature assessment, tissue-expression data, KEGG pathway enrichment analysis and DisGeNET disease-association scores. Created with BioRender.com.

### 2.1 Curation of GO terms

Two states of interest (SOIs) were defined based on the research objective: osteoporosis and sarcopenia. A curated list of GO terms representing the biological processes relevant to osteoporosis and sarcopenia was generated following a systematic workflow adapted from Škorjanc et al. (Škorjanc et al., 2025). The curation process began with an extensive literature review to delineate key pathophysiology for each condition (e.g., “endocrine and hormonal regulation of bone”). Each mechanistic group was then translated into a representative keyword (e.g., “estrogen”) for targeted manual searches within the QuickGO database, selecting primarily GO terms within the biological process sub-ontology of GO.

Terms were included when they: (i) described a biological process plausibly relevant to osteoporosis, sarcopenia or shared bone–muscle biology; (ii) represented a sufficiently specific mechanism, cell type or regulatory process; and (iii) could be assigned a biologically interpretable direction with respect to the corresponding SOI. To reduce excessive generality and redundancy, GO terms nested deeper within the GO term redundancy tree, describing more specific biological concepts (i.e., “child terms”) were preferred over broader, less-specific “parent terms” when both represented the same biological concept. Stimulatory terms were defined as biological processes expected to promote or exacerbate the disease-relevant state, whereas inhibitory terms were defined as processes expected to oppose or reduce that state. For processes with established context-dependent or bidirectional biological effects, a term could be retained in both directional categories when the process was considered relevant to the SOI but could not be represented adequately by a single direction. For example, parathyroid hormone-related processes were retained as context-dependent hormonal mechanisms because prolonged PTH excess and intermittent therapeutic PTH-receptor stimulation may exert opposing effects on bone remodelling.

The curated list was reviewed by two researchers with experience in bone and muscle biology. Formal inter-rater agreement was not recorded during the original curation process. Disagreements regarding term inclusion, SOI assignment or directionality were resolved by consensus. A summary of the curated GO-term categories, SOI assignments and stimulatory/inhibitory directions is provided in Supplementary Material 4 (Supplementary Tables S1,S2), while the complete term-level input list is provided in Supplementary Material 1.

### 2.2 Reverse enrichment analysis

Reverse enrichment analysis was performed using GOReverseLookup (v1.0.56; Python 3.11) on 27 June 2025. The primary analysis targeted *Homo sapiens*, incorporating ortholog data from *Danio rerio*, *Rattus norvegicus*, *Mus musculus*, and *Xenopus tropicalis*. Key parameters included the use of indirect annotations up to three parent levels and the exclusion of electronically inferred annotations (IEA). To ensure reproducibility, all input files are made readily available at (https://github.com/GenomeDev/GOReverseLookup/tree/main/research_models). We used the Gene Ontology OBO (format-version 1.2; data-version releases/2025–03-16), the *H. sapiens* UniProt-GOA annotation set (GO Annotations File version 2.2; date-generated 2024–06-19), and GO metadata via the GO API (release 2025–06-26). Orthologs to human (NCBITaxon:9606) were mapped from mouse (MGI; 10,090), rat (RGD; 10,116), zebrafish (ZFIN; 7955), and tropical clawed frog (Xenbase; 8364).

GOReverseLookup evaluates the association between each gene product and a SOI independently by determining whether a gene product is annotated with GO terms describing a SOI more often than would be expected relative to its remaining GO annotations across the entire universe of GO terms in existence. These associations are evaluated using the Fisher’s exact test followed by Benjamini–Hochberg false discovery rate correction. The primary threshold of *q* < 5 × 10^−8^ was selected to define a highly conservative core candidate set, motivated by the stringent significance criterion used in the initial GOReverseLookup proof-of-concept applications. Because the present study applies this criterion to BH-adjusted *q*-values and the threshold is considerably more stringent than conventional FDR criteria, additional sensitivity analyses were performed at *q* < 0.01 and *q* < 0.05. To evaluate dependence on ortholog-derived annotation coverage, the analysis was repeated under three annotation configurations while retaining the original GO-term input model and all other analytical parameters: (i) human-only annotations; (ii) human annotations supplemented with ortholog-derived annotations from mouse and rat; and (iii) the primary full cross-species model additionally incorporating ortholog-derived annotations from zebrafish and *X. tropicalis*.

### 2.3 Systematic characterization of candidate genes

Candidate genes prioritized by GOReverseLookup were further characterized through a systematic three-step contextual assessment process.

#### 2.3.1 Literature and database-based disease association assessment

A comprehensive literature search was performed in PubMed and Google Scholar to identify existing evidence linking each gene to the diseases of interest. Search queries combined the gene symbol with the keywords “osteoporosis,” “sarcopenia,” and “osteosarcopenia.” In parallel, gene-disease associations were quantified using the DisGeNET database version 25.1.1. For each gene, we recorded the evidence-weighted Gene-Disease Association (GDA) score, which aggregates contributions from expert-curated human sources (0.30 each), animal-model databases (0.10 each) and PubMed text-mining hits (small, cumulative weights). Scores range from 0 to 1, with values greater than 0.30 indicating support from at least one expert-curated human source (Piñero et al., 2015). The primary objective of this step was to determine whether a gene had been previously associated with either osteoporosis, sarcopenia, or their co-manifestation (osteosarcopenia) in the scientific literature or curated databases.

#### 2.3.2 Tissue-specific expression

Gene expression levels in disease-relevant human tissues were assessed using the Expression Atlas (EMBL-EBI). For each gene, the highest recorded expression level was extracted across muscle-related tissues (“muscle of arm,” “muscle of leg,” “skeletal muscle,” “skeletal muscle of trunk,” and “skeletal muscle tissue”) and bone tissue (“cervical vertebrae”). Expression levels were categorized by transcripts per million (TPM) as high (>1000), medium (>11), low (>0.5), or below cutoff (≤0.5 TPM), using thresholds adopted from the Expression Atlas baseline expression classification. It should be noted that bone tissue datasets in Expression Atlas are limited in diversity and coverage (https://www.ebi.ac.uk/gxa/experiments?species=%22homo%20sapiens%22).

#### 2.3.3 Pathway enrichment analysis

To identify enriched biological pathways, KEGG (Kyoto Encyclopedia of Genes and Genomes) enrichment pathway analysis was performed using ShinyGO v0.82 (https://bioinformatics.sdstate.edu/go/). The background reference gene set was defined as all human protein-coding genes annotated in the KEGG database, and enrichment was calculated using the hypergeometric test (default ShinyGO settings). Pathways were ranked according to fold enrichment (FE) and considered significantly enriched at a Benjamini–Hochberg-adjusted *p*-value (*q*) < 5 × 10^−5^.

## 3 Results

### 3.1 Identification and categorization of candidate genes

In the primary full cross-species analysis, the population set comprised 30,768 GO terms. Statistical scoring was initiated for 25,504 gene-product records, of which 2,461 had sufficient eligible annotations to yield Fisher-test results for all four directional SOIs: stimulation and inhibition of osteoporosis and sarcopenia. Gene-product records without eligible GO annotations could not be prioritized.

Our primary full cross-species analysis prioritized 37 candidate genes with significant enrichment for GO annotations related to both curated osteoporosis- and sarcopenia-related states of interest (*q* < 5 × 10^−8^). To prioritize these candidates for further follow-up analysis, we classified them according to prior literature evidence and tissue-specific expression patterns in bone and skeletal muscle (Figure 2). Comprehensive statistical outputs are provided in Supplementary Material 2.

**FIGURE 2:**
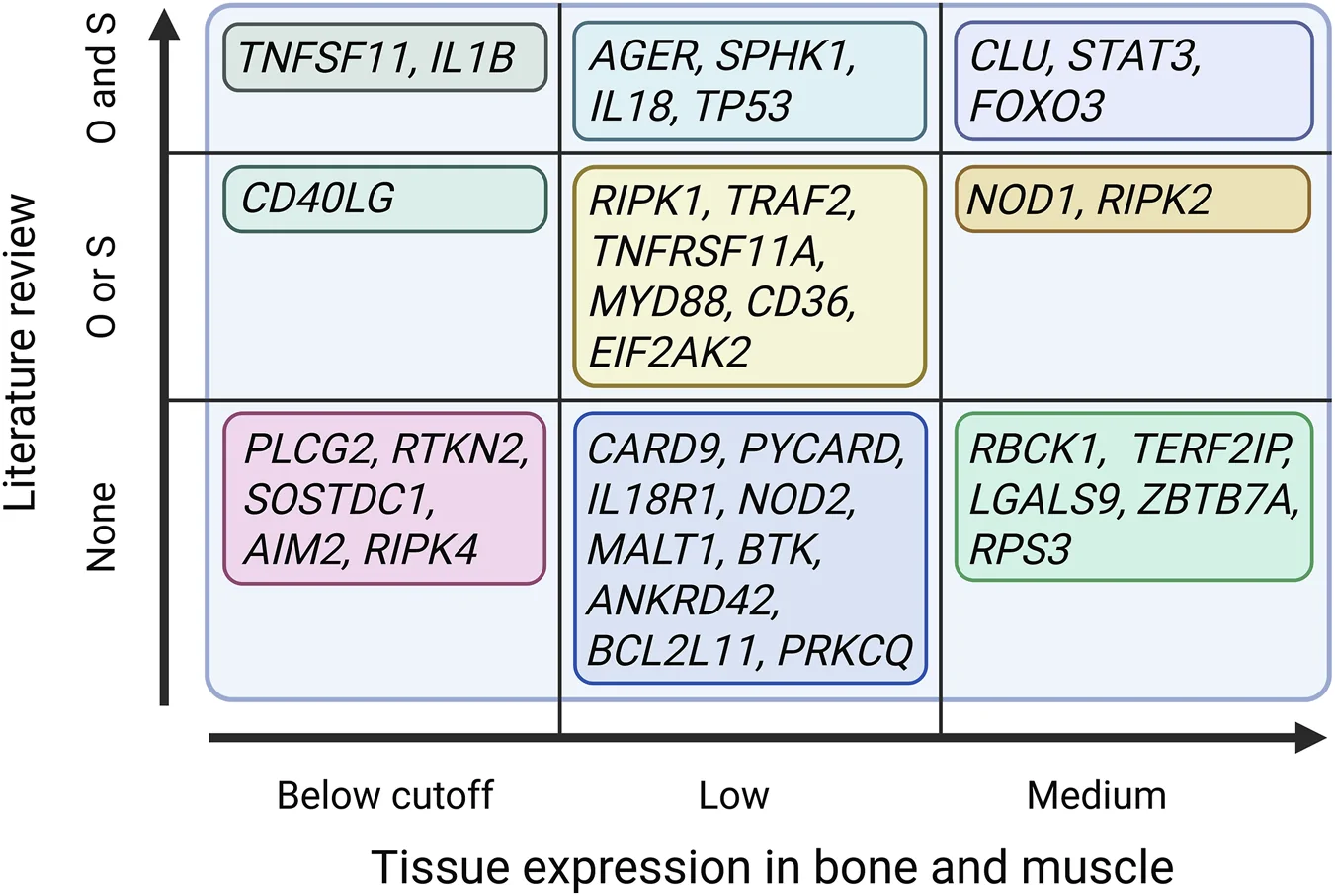
Prioritized candidate genes categorized according to targeted literature evidence and tissue expression. The 37 candidate genes prioritized in the primary GOReverseLookup analysis were categorized using a targeted literature assessment performed in PubMed and Google Scholar. The y-axis denotes literature-based evidence identified through this assessment: None - no prior association with either osteoporosis or sarcopenia identified in the targeted search; O or S - prior association identified with osteoporosis or sarcopenia; and O and S -prior association identified with both conditions. Tissue-expression data were obtained from the EMBL-EBI Expression Atlas. The x-axis categorizes expression in the selected bone- and muscle-related datasets as Below cutoff (<0.5 TPM), Low (>0.5 to 11 TPM), or Medium (>11 to 1000 TPM).

The analysis highlighted 37 candidate genes, including 14 genes with no previously reported association to either disease, nine genes previously described in the literature as associated with both osteoporosis and sarcopenia and with non-zero GDA scores for these conditions, and 14 significant genes showing either evidence linked to only one of the two conditions or expression levels below the defined cutoff in one or both tissue types. Among the 14 genes with no previously reported association, five genes (*RBCK1, TERF2IP, LGALS9, ZBTB7A,* and *RPS3*) exhibited at least moderate (medium) expression in both bone and muscle and no existing disease association score (GDA ≈0), whereas the remaining nine genes (*PYCARD, MALT1, BCL2L11, PRKCQ, CARD9, IL18R1, ANKRD42, BTK, NOD2*) displayed detectable but lower (low) expression in both tissues. Among the nine previously described genes *(TNFSF11, STAT3, IL1B, AGER, CLU, SPHK1, IL18, TP53, FOXO3),* two genes, *TNFSF11* and *STAT3*, have been directly implicated in osteosarcopenia (Jang and Choi, 2024; Liu et al., 2024).

### 3.2 Sensitivity analyses for significance threshold and ortholog-derived annotations

To assess the influence of the stringent primary BH-FDR threshold and cross-species annotation propagation, we repeated the prioritization analysis across three FDR thresholds and three annotation configurations. The primary full cross-species model prioritized 37 gene candidates, compared with 208 candidate genes at q < 0.01 and 200 candidate genes at q < 0.05 (Table 1). The lower number of candidates at q < 0.05 than at q < 0.01 reflects the bidirectional prioritization rule, in which genes could be excluded if they showed significant association with the negative state of interest. The human-only model yielded no prioritized candidates at any of the assessed BH-FDR thresholds. At the primary threshold of q < 5 × 10^−8^, inclusion of mammalian ortholog-derived annotations from mouse and rat yielded 31 prioritized candidates, of which 28 were retained from the primary 37-gene full cross-species candidate set. Ten of the 14 less-characterized candidates were retained in the mammalian-expanded model; the four not retained were *BCL2L11, BTK, IL18R1* and *RBCK1*. Complete gene-level outputs for all nine sensitivity-analysis configurations are provided in Supplementary Material 5.

**TABLE 1:** Sensitivity of candidate-gene prioritization to ortholog-derived annotation inclusion and BH-FDR threshold.

Species contributing GO annotations	*q* < 5 × 10^−8^	*q* < 0.01	*q* < 0.05
*H. sapiens*	0	0	0
*H. sapiens* + *M. musculus + R. norvegicus*	31	186	188
*H. sapiens* + *M. musculus* + *R. norvegicus* + *D. rerio* + *X. tropicalis* (primary analysis)	37	208	200

Candidate genes were prioritized when they met the corresponding BH-FDR, threshold for enrichment of GO, annotations related to both curated states of interest. The full cross-species model at *q* < 5 × 10^−8^ represents the predefined primary analysis and generated the primary 37-gene candidate set. All sensitivity analyses used the same original GO-term input model, indirect-annotation depth and evidence-code filtering, only the included ortholog-derived annotation sources and/or BH-FDR, threshold were varied.

### 3.3 KEGG pathway analysis

KEGG enrichment of the 37 prioritized candidate genes yielded 15 significantly overrepresented pathways that exceeded the predefined significance threshold (*q* < 5 × 10^−5^) and showed a fold enrichment (FE) higher than 25. To emphasize biological mechanisms rather than incidental annotation overlap, only non-infectious pathways are presented in Table 2. The complete list, including infection-related pathways (e.g., Malaria, Shigellosis, Tuberculosis), is provided in Supplementary Material 3. Pathways are ranked by FE, the effect-size metric recommended by ShinyGO for assessing biological relevance. In a sensitivity analysis excluding the highly connected immune-associated genes *IL1B, IL18, MYD88, PYCARD, RIPK1, TRAF2, NOD2*, and *BTK*, no KEGG pathways met the predefined combined thresholds of *q* < 5 × 10^−5^ and FE > 25.

**TABLE 2:** KEGG pathways enriched among the 37 prioritized candidate genes.

Pathways	FE	*q*	Genes
NF-kappa B signaling	57.9	4.9 × 10^−14^	*MALT1, IL1B, MYD88, PLCG2, PRKCQ, BTK, TRAF2, TNFSF11, RIPK1, TNFRSF11A*
Cytosolic DNA-sensing pathway	47.8	7.6 × 10^−7^	*PYCARD, IL1B, IL18, RIPK1, AIM2*
NOD-like receptor signaling pathway	39.5	7.7 × 10^−15^	*NOD1, RBCK1, PYCARD, IL1B, IL18, MYD88, NOD2, CARD9, TRAF2, RIPK1, RIPK2, AIM2*
Inflammatory bowel disease	37.1	3.1 × 10^−5^	*IL1B, IL18, NOD2, STAT3*
Prolactin signaling pathway	34.4	3.9 × 10^−5^	*FOXO3, STAT3, TNFSF11, TNFRSF11A*
C-type lectin receptor signaling pathway	28.9	8.7 × 10^−6^	*MALT1, PYCARD, IL1B, PLCG2, CARD9*
Osteoclast differentiation	28.7	7.6 × 10^−7^	*IL1B, PLCG2, BTK, TRAF2, TNFSF11, TNFRSF11A*
TNF signaling pathway	26.9	1.2 × 10^−5^	*IL1B, NOD2, TRAF2, RIPK1, IL18R1*
Necroptosis	26.5	1.3 × 10^−7^	*RBCK1, PYCARD, IL1B, EIF2AK2, STAT3, TRAF2, RIPK1*
Lipid and atherosclerosis	25.3	1.7 × 10^−9^	*PYCARD, IL1B, IL18, MYD88, STAT3, TP53, TRAF2, CD36, CD40LG*

FE, fold enrichment; *q* - Benjamini–Hochberg–adjusted *p*-value. Pathways were identified with ShinyGO, v0.82 using the KEGG, *Homo sapiens* gene universe (hypergeometric enrichment test, default settings). Results are ordered by FE, and limited to pathways with FE > 25 and Benjamini–Hochberg-adjusted *p-*values (*q*) < 5 × 10^−5^. Infection-related pathways that also met the significance threshold (e.g., *Malaria, Shigellosis, Tuberculosis*) are presented in Supplementary Material 3.

## 4 Discussion

This study represents the first integrative application of reverse Gene Ontology analysis to a complex musculoskeletal phenotype. Our analysis prioritized 37 genes enriched for annotations related to both curated osteoporosis- and sarcopenia-related states of interest, systematically categorized by prior disease associations, expression across bone and skeletal muscle, and their pathway context derived from KEGG enrichment. This integrative framework recovered several genes previously linked to bone and muscle biology, supporting the biological plausibility and face validity of the prioritization approach. Incorporating tissue-specific expression further refines gene prioritization, as genes expressed in both bone and muscle are more likely to influence shared metabolic and inflammatory signaling axes that maintain musculoskeletal integrity. However, genes with relatively low expression but strong biological relevance, such as *TNFSF11* and *IL1B*, indicate that expression data should be interpreted alongside known cell-type specificity, inducible expression, and pathway function rather than by quantitative thresholds alone.

Genes with limited prior association to either osteoporosis or sarcopenia represent particularly valuable, underexplored candidates that may illuminate biological mechanisms not yet captured by current literature. Five such genes, *RBCK1, TERF2IP, LGALS9, ZBTB7A,* and *RPS3*, showed moderate expression in both bone and skeletal muscle, suggesting a possible contribution to the shared molecular and inflammatory networks underpinning osteosarcopenia.

*RBCK1* encodes HOIL-1, a catalytic component of the linear ubiquitin chain assembly complex (LUBAC), one of the three subunits responsible for M-1–linked ubiquitination (Guo et al., 2023). LUBAC regulates multiple immune signaling cascades, particularly through activation of the NF-κB pathway (Jahan et al., 2021), a mechanism consistently enriched in our KEGG analysis. Beyond inflammation, HOIL-1 also modulates apoptosis and necroptosis (Ju et al., 2022), two processes essential for tissue remodeling in both muscle and bone. Mutations in *RBCK1* cause RBCK1-associated polyglucosan body myopathy-1 (PGBM1), a glycogen storage disorder that manifests with skeletal muscle weakness and cardiomyopathy (Pühringer et al., 2024), further supporting its potential relevance to musculoskeletal pathology.

Similarly, *LGALS9* encodes Galectin-9, a β-galactoside-binding lectin with broad immunomodulatory functions (Lv et al., 2022). In bone, Galectin-9 suppresses osteoclastogenesis through the TIM-3 receptor and promotes osteoblast differentiation (Moriyama et al., 2014; Russo et al., 2025), thereby contributing to balanced bone turnover. Galectin-9 also restrains the NLRP3 inflammasome by binding to NLRP3 and inducing its p62-dependent autophagic degradation (Wang et al., 2021), a process consistent with the enrichment of innate immune and cytosolic DNA-sensing pathways identified in this study. Elevated Galectin-9 levels have further been reported as a biomarker of frailty (Mitchell et al., 2023), reinforcing its potential involvement in systemic processes coupling bone and muscle loss.

The *TERF2IP* gene encodes Rap1 (Repressor/Activator Protein-1), a shelterin complex component that safeguards telomeric DNA integrity (Deregowska and Wnuk, 2021). In addition to its structural telomere role, Rap1 acts as an adaptor within the IκB kinase (IKK) complex, amplifying canonical NF-κB signaling and driving pro-inflammatory gene expression (Teo et al., 2010). Phosphorylation-dependent nuclear export of Rap1, or telomere dysfunction, triggers a DNA-damage response and cellular senescence, accompanied by a senescence-associated secretory phenotype (SASP) enriched in cytokines such as IL-6 and TNF-α (Kotla et al., 2019). These cytokines are well-known mediators of osteoclast activation and muscle proteolysis, providing a plausible mechanistic bridge between telomere stress, inflammation, and osteosarcopenic remodeling.

*ZBTB7A* encodes the leukemia/lymphoma-related factor (LRF, also known as Pokémon), a zinc-finger transcription factor that orchestrates lineage-specific differentiation (Tsuji-Takechi et al., 2012). Within the osteoclast lineage (originally cloned as OCZF), ZBTB7A exerts stage-specific effects: repressing early RANKL-driven commitment by limiting NFATc1 induction, while in mature osteoclasts supporting resorptive activity and cell survival (Tsuji-Takechi et al., 2012; Zou et al., 2022). In skeletal muscle, *in-vitro* studies indicate that suppression of *ZBTB7A* by miR-182–5p facilitates myogenic differentiation (Zhang M. et al., 2025), implying a context-dependent regulatory function relevant to both tissue types.

Finally, *RPS3* encodes ribosomal protein S3, a component of the 40S small ribosomal subunit with multiple extra-ribosomal roles, including regulation of apoptosis, DNA repair, and transcription (Kim et al., 2016). RPS3 also acts as a noncanonical subunit of the NF-κB complex, directly modulating the transcription of specific target genes (Wan et al., 2007; García-García et al., 2021). As NF-κB activity governs both osteoclastogenesis and catabolic inflammation in aging muscle, *RPS3* may represent a nodal element linking these processes. Moreover, ribosomal biogenesis is a key determinant of protein synthesis capacity; its impairment in aging muscle contributes to anabolic resistance, although data on *RPS3* expression in sarcopenic tissue remain limited (Chaillou and Montiel-Rojas, 2023).

Conversely, recovery of two genes previously discussed in relation to osteosarcopenia and nine additional genes previously linked to both osteoporosis and sarcopenia provide evidence that the prioritization framework captures established annotation-supported biology. Importantly, the presence of prior evidence does not reduce their relevance within the combined osteosarcopenic phenotype but rather highlights convergent mechanisms across the bone–muscle axis.

Among these, *TNFSF11* is the most translationally advanced example. Its encoded ligand, RANKL, is a key driver of osteoclast differentiation and the established pharmacological target of denosumab therapy. While several small and heterogeneous clinical studies have suggested potential improvements in muscle performance following denosumab treatment (Rupp et al., 2022), a recent randomized controlled trial reported no significant difference in muscle health between denosumab and placebo groups (Haeri et al., 2025). Conversely, *STAT3* emerges from transcriptomic analyses as a shared molecular hub for both postmenopausal osteoporosis and sarcopenia (Liu et al., 2024). Although this points to *STAT3* as a promising therapeutic target for dual bone–muscle interventions, experimental validation across both tissue systems remains limited.

Two other genes, *FOXO3* and *AGER*, though less explored in the combined phenotype, have been independently implicated in each disorder. *FOXO3* is a central regulator of cellular aging and oxidative stress (Zhang L. et al., 2025). In skeletal muscle, it acts downstream of the IGF-1/PI3K/AKT pathway, where nuclear accumulation of *FOXO3* correlates with muscle atrophy and sarcopenic phenotype (Gellhaus et al., 2023). In bone, reduced *FOXO3* expression is characteristic of osteoporotic patients, and *FOXO3* deficiency exacerbates oxidative damage and bone loss in aged mice (Zhang W. et al., 2025).

*AGER*, encoding the receptor for advanced glycation end products (RAGE), is similarly positioned at the intersection of inflammation, metabolism, and aging (Zhang et al., 2023). Activation of RAGE by AGEs stimulates NF-κB signaling, thereby promoting catabolic processes and muscle cell loss (Riuzzi et al., 2012; Zhang et al., 2023). Osteoporosis has also been proposed to partially mediate the association between circulating AGEs and sarcopenia (Zhang et al., 2023). In bone, RAGE signaling suppresses osteoblast proliferation and enhances osteoclastogenesis, driving the osteoporotic phenotype (Zhou et al., 2023). Collectively, these established genes demonstrate that the framework recovers biologically plausible candidates within previously annotated bone–muscle mechanisms.

KEGG pathway enrichment revealed convergence around three interrelated mechanistic themes: inflammatory signalling integrated with osteoclast regulation, innate immune sensing with inflammasome-associated cytokine signalling, and inflammatory stress-associated regulated cell-death responses. The enrichment of NF-κB signalling, TNF signalling, and osteoclast differentiation was driven in part by genes including *TNFSF11, TNFRSF11A, PLCG2, BTK, TRAF2, RIPK1*, and *IL1B*. This pattern is consistent with the close biological relationship between inflammatory signalling and osteoclast regulation, particularly through RANKL–RANK-associated mechanisms. These findings may also be relevant to skeletal muscle loss, in which inflammatory and catabolic signalling can contribute to impaired muscle homeostasis (Reed et al., 2011; Gostage et al., 2024).

A second theme involved innate immune sensing and inflammasome-associated cytokine signalling. The enrichment of NOD-like receptor signalling, cytosolic DNA-sensing, and C-type lectin receptor signalling was supported by overlapping genes including *NOD1, NOD2, RIPK2, AIM2, PYCARD, IL1B, IL18, MYD88*, and *CARD9*. In particular, the cytosolic DNA-sensing pathway was enriched through *AIM2, PYCARD, IL1B, IL18*, and *RIPK1*, indicating an AIM2/inflammasome-associated inflammatory signal within the candidate set. Thus, this enrichment is most appropriately interpreted as evidence of a broader sterile innate immune and inflammasome-related theme.

A third related theme involved inflammatory stress-associated regulated cell-death signalling, represented by enrichment of the necroptosis pathway through *RBCK1, PYCARD, IL1B, EIF2AK2, STAT3, TRAF2*, and *RIPK1*. Necroptosis is a regulated form of lytic cell death mediated primarily through *RIPK1, RIPK3*, and *MLKL* (Hu et al., 2022). Its biological relevance to musculoskeletal decline is supported by preclinical studies implicating necroptosis-related signalling in bone pathology, including glucocorticoid-induced osteoporosis (Feng et al., 2014; Hu et al., 2022), and by experimental evidence that inhibition of necroptosis-related signalling can reduce muscle-cell death, inflammation, and weakness in inflammatory myopathy models (Kamiya et al., 2022). In the present analysis, the overlap between necroptosis, TNF signalling, and innate immune pathways suggests that regulated cell-death mechanisms may form part of a broader inflammatory tissue-stress response relevant to concurrent bone and muscle deterioration.

Although several infection-related KEGG terms (e.g., *Malaria*, *Shigellosis*, *Tuberculosis*, *Legionellosis*) also surpassed the enrichment threshold, these associations were primarily driven by broad innate immunity genes, including *IL1B*, *IL18*, *MYD88*, *PYCARD*, *RIPK1*, *TRAF2*, and *NOD2,* reflecting generalized inflammasome and NF-κB signalling rather than pathogen-specific mechanisms. Their co-enrichment further underscores the central role of innate immune pathways in the systemic regulation of musculoskeletal homeostasis.

A major strength of GOReverseLookup lies in its ability to let researchers explicitly define states of interest by curating GO terms that reflect disease-relevant mechanisms, thereby producing transparent, auditable, and biologically interpretable gene lists. In contrast to black-box artificial intelligence models, GOReverseLookup offers full methodological transparency. Users can inspect the queried processes, inclusion criteria, and resulting statistical associations. The inclusion of ortholog-aware querying (via Ensembl and UniProtKB) expands annotation coverage and helps surface evolutionarily conserved or understudied candidate genes while preserving reproducibility. Importantly, the tool performs particularly well for composite or multifactorial phenotypes, such as osteosarcopenia, where intersecting GO term sets capture biologically meaningful overlaps between related conditions.

Nevertheless, several methodological limitations should be acknowledged. Because the published literature preferentially reflects the most extensively studied biological mechanisms and their associated genes, using literature-derived GO terms as input for GOReverseLookup may introduce circularity. Specifically, well-characterized genes are more comprehensively annotated and may therefore be more likely to be recovered by the analysis. Careful literature curation and transparent GO-term selection are therefore essential to reduce literature and annotation bias. However, the potential for circularity cannot be fully eliminated and should be considered when interpreting the results, especially when established disease-related genes are identified. Moreover, because candidate prioritization and subsequent biological interpretation rely on overlapping annotation-based resources, including GO, KEGG, and DisGeNET, the recovery of established disease-associated genes cannot be considered independent validation of the framework. The robustness of GOReverseLookup outcomes critically depends on the careful selection and curation of GO terms, as well as on the chosen analytical parameters. Suboptimal input curation may yield biologically irrelevant or misleading associations. While cross-species ortholog integration expands candidate coverage, it can also increase the risk of false positives. Furthermore, the accuracy of the results is inherently constrained by the completeness of existing GO annotations; insufficient or outdated annotation data may lead to false negatives.

The sensitivity analysis of orthologous genes provides an important qualification of the primary findings. No candidates were prioritized using human annotations alone under the stringent primary threshold (5 × 10^−8^), but the majority of primary candidates were retained after inclusion of mouse and rat ortholog-derived annotations. Ortholog propagation may capture conserved biological processes that are incompletely annotated in humans, but it may also introduce annotations derived from species-specific developmental, immune or experimental contexts. The lower number of candidates at q < 0.05 than at q < 0.01 further reflects that the prioritization procedure incorporated both positive and negative associations with the state of interest. Therefore, a more relaxed threshold may exclude additional genes if their association with the negative state of interest becomes significant. Accordingly, the candidate list should be treated as an ortholog-informed hypothesis resource requiring validation in human datasets or disease-relevant human experimental models.

The availability of tissue-specific expression datasets also presents a limitation, particularly for bone tissue. In the present analysis, the only available bone-related dataset was cervical vertebral tissue, whereas substantially richer datasets were available for skeletal muscle. Cervical vertebral samples represent a heterogeneous mixture of cell types and may not adequately capture the expression profiles of specific bone-relevant populations, including osteoblasts, osteocytes, osteoclasts, bone marrow mesenchymal stromal cells, stromal cells, and immune cells. Moreover, gene-expression profiles may differ across skeletal sites and bone compartments, meaning that cervical vertebral tissue may not fully represent bone tissue more broadly or clinically relevant sites affected in osteoporosis and osteosarcopenia. Similarly, skeletal muscle datasets may include signals from myofibers, satellite cells, fibro-adipogenic progenitors, stromal cells, vascular cells, and immune populations, all of which may be biologically relevant in osteosarcopenia. Accordingly, the present analysis provides tissue-level evidence of candidate-gene expression and should not be interpreted as resolving cell-type-specific expression patterns.

Regarding KEGG pathway analysis, we observed strong enrichment of innate immune and inflammatory pathways. This may partly reflect annotation and literature bias, as broadly studied inflammatory mediators, including *IL1B, IL18, MYD88, PYCARD, RIPK1, TRAF2, NOD2*, and *BTK*, are extensively represented across multiple KEGG pathways. In a sensitivity analysis excluding these eight immune-associated genes, no pathway met the predefined combined thresholds. This suggests that the primary KEGG enrichment profile was substantially influenced by broadly annotated innate immune and inflammatory genes. Consequently, pathway-level enrichment may preferentially emphasize generic inflammatory signalling rather than mechanisms more specific to bone–muscle interactions in osteosarcopenia. Nevertheless, chronic low-grade inflammation is considered an important pathophysiological mechanism underlying osteosarcopenia and may therefore represent a biologically meaningful component of the observed enrichment. A degree of circularity may also exist because inflammatory pathways are strongly represented both in the source literature used for GO-term selection and in downstream pathway databases.

The present study is also limited by the absence of experimental validation and by the lack of validation in independent patient cohorts or external genetic resources, such as GWAS or TWAS datasets. In addition, sex-stratified analyses were not performed, although osteoporosis, sarcopenia, and osteosarcopenia show important sex-specific epidemiological and potentially biological differences. Finally, the present annotation-based prioritization framework cannot distinguish causal drivers of osteosarcopenia from associated biomarkers or downstream consequences of inflammation and tissue degeneration. The identified genes should therefore be interpreted as prioritized candidates requiring further independent validation rather than as confirmed biomarkers or causal determinants of osteosarcopenia.

A logical next step in osteosarcopenia research would be to use the candidate gene list generated by GOReverseLookup as a hypothesis-generating resource for further validation. In practice, researchers may first prioritize a smaller subset of genes supported by multiple lines of evidence, including GO-based prioritization, disease relevance, tissue-expression patterns, and biological plausibility. Depending on the availability of suitable data and biological material, these candidates could subsequently be examined in independent transcriptomic or genetic datasets, patient-derived samples, or experimental models relevant to bone and muscle biology.

## Data Availability

The original contributions presented in the study are included in the article/Supplementary Material, further inquiries can be directed to the corresponding author.
