# Supplementary material for: Bridging bone and muscle: ontology-driven prioritization of candidate genes at the osteoporosis–sarcopenia interface by gene ontology reverse lookup
Source: Front Genet. 2026 Sep 25;17:1760184. doi: 10.3389/fgene.2026.1760184 (PMC13649218; doi:10.3389/fgene.2026.1760184)
Supplement: Supplementary file 1 [file Table2.docx]

**Supplementary material 2**: **Full statistical output of GOReverseLookup and analysis of genes**.

| Gene | *q*-value (O) | *q*-value (S) | Literature (O) | Literature (S) | GDA (O) | GDA (S) | Expression in bone | Expression in muscle |
| --- | --- | --- | --- | --- | --- | --- | --- | --- |
| *NOD1* | 7.31e-19 | 1.00e-21 | [1] | / | 0.1 | 0 | medium | medium |
| *PLCG2* | 6.73e-17 | 2.37e-17 | / | / | 0 | 0 | below cutoff | low |
| *RBCK1* | 3.88e-21 | 1.04e-16 | / | / | 0 | 0 | medium | medium |
| *RIPK1* | 5.73e-37 | 9.26e-16 | [2] | / | 0 | 0 | low | medium |
| *CARD9* | 2.21e-21 | 1.46e-15 | / | / | 0 | 0 | low | low |
| *RTKN2* | 1.65e-15 | 5.48e-15 | / | / | 0 | 0 | below cutoff | low |
| *PYCARD* | 5.49e-26 | 4.71e-14 | / | / | 0 | 0 | low | low |
| *IL18R1* | 2.13e-14 | 6.57e-14 | / | / | 0 | 0 | low | low |
| *RIPK2* | 1.53e-13 | 2.15e-14 | [1] | / | 0 | 0 | medium | medium |
| *TNFRSF11A* | 3.26e-31 | 2.16e-13 | [3] | / | 0.85 | 0 | low | medium |
| *MYD88* | 1.20e-16 | 5.07e-13 | [4] | / | 0 | 0.15 | low | medium |
| *AGER* | 2.60e-22 | 5.73e-13 | [5] | [6] | 0.3 | 0.3 | low | medium |
| *TNFSF11* | 1.34e-35 | 8.05e-13 | [7] | [7] | 1 | 0.1 | no data | low |
| *TERF2IP* | 1.32e-11 | 3.31e-14 | / | / | 0.1 | 0.1 | medium | medium |
| *CLU* | 4.61e-12 | 1.94e-11 | [8] | [9] | 0.2 | 0 | medium | medium |
| *CD36* | 6.86e-16 | 2.89e-11 | [10] | / | 0.1 | 0 | low | medium |
| *STAT3* | 1.66e-31 | 7.56e-11 | [11] | [11] | 0.3 | 0.1 | medium | medium |
| *NOD2* | 6.53e-22 | 3.15e-10 | / | / | 0.2 | 0 | low | low |
| *SOSTDC1* | 3.33e-10 | 1.08e-11 | / | / | 0 | 0 | below cutoff | medium |
| *EIF2AK2* | 1.09e-16 | 4.11e-10 | [12] | / | 0.2 | 0 | low | medium |
| *IL1B* | 4.23e-10 | 4.94e-16 | [13] | [14] | 0.5 | 0.25 | low | below cutoff |
| *CD40LG* | 1.35e-10 | 3.86e-10 | [15] | / | 0.2 | 0 | below cutoff | low |
| *LGALS9* | 1.60e-23 | 1.70e-09 | / | / | 0 | 0 | medium | medium |
| *MALT1* | 5.09e-10 | 1.41e-09 | / | / | 0.2 | 0 | low | medium |
| *AIM2* | 7.93e-10 | 2.23e-09 | / | / | 0 | 0 | below cutoff | below cutoff |
| *SPHK1* | 3.41e-09 | 6.39e-14 | [16] | [17] | 0 | 0 | low | low |
| *BTK* | 9.48e-10 | 2.67e-09 | / | / | 0 | 0 | low | low |
| *ZBTB7A* | 1.04e-15 | 3.71e-09 | / | / | 0 | 0 | medium | medium |
| *TRAF2* | 1.04e-09 | 2.90e-09 | [18] | / | 0 | 0 | low | medium |
| *IL18* | 4.75e-09 | 1.03e-13 | [19] | [20] | 0.2 | 0.7 | medium | low |
| *ANKRD42* | 1.92e-09 | 3.50e-09 | / | / | 0 | 0 | low | low |
| *TP53* | 9.27e-17 | 9.32e-09 | [21] | [22] | 0.3 | 0.2 | low | medium |
| *FOXO3* | 2.71e-09 | 7.62e-09 | [23] | [24] | 0.25 | 0.2 | medium | medium |
| *BCL2L11* | 3.36e-09 | 8.49e-09 | / | / | 0 | 0 | low | medium |
| *RPS3* | 3.33e-14 | 2.08e-08 | / | / | 0 | 0 | medium | medium |
| *RIPK4* | 1.62e-08 | 3.00e-08 | / | / | 0 | 0 | below cutoff | below cutoff |
| *PRKCQ* | 1.40e-08 | 3.97e-08 | / | / | 0 | 0 | low | medium |

Table note: O - osteoporosis; S - sarcopenia; GDA - gene–disease association score (DisGeNET v25.1.1). Values are two-tailed enrichment *p*-values adjusted for multiple testing with the Benjamini–Hochberg procedure (*q*); genes are shown if significant in both condition at *q* < 5 × 10⁻⁸. The Literature columns provide numbered citations supporting osteoporosis and/or sarcopenia links identified in the targeted search. The GDA collums provide DisGeNET gene–disease association scores. Expression levels in bone and muscle (Expression Atlas, EMBL-EBI) are categorized by TPM: High (>1000), Medium (>11), Low (>0.5), Below cutoff (≤0.5).

[1]	J. Wei, Q. Liu, H.-Y. Yuen, A.C.-H. Lam, Y. Jiang, Y. Yang, Y. Liu, X. Zhao, L. Xiao, Gut-bone axis perturbation: Mechanisms and interventions via gut microbiota as a primary driver of osteoporosis, J. Orthop. Transl. 50 (2025) 373–387. https://doi.org/10.1016/j.jot.2024.11.003.

[2]	X. Hu, Z. Wang, C. Kong, Y. Wang, W. Zhu, W. Wang, Y. Li, W. Wang, S. Lu, Necroptosis: A new target for prevention of osteoporosis, Front. Endocrinol. 13 (2022) 1032614. https://doi.org/10.3389/fendo.2022.1032614.

[3]	M. McClung, Role of RANKL inhibition in osteoporosis, Arthritis Res. Ther. 9 (2007) S3. https://doi.org/10.1186/ar2167.

[4]	J. Alshaweesh, R. Dash, M.S.J. Lee, P. Kahyaoglu, E. Erci, M. Xu, J. Matsuo-Dapaah, C. Del Rosario Zorrilla, K. Aykac, S. Ekemen, K. Kobiyama, K.J. Ishii, C. Coban, MyD88 in osteoclast and osteoblast lineages differentially controls bone remodeling in homeostasis and malaria, Int. Immunol. 36 (2024) 451–464. https://doi.org/10.1093/intimm/dxae023.

[5]	J. Zhou, S. Liu, S. Bi, W. Kong, R. Qian, X. Xie, M. Zeng, X. Jiang, Z. Liao, M. Shuai, W. Liu, L. Cheng, M. Wu, The RAGE signaling in osteoporosis, Biomed. Pharmacother. Biomedecine Pharmacother. 165 (2023) 115044. https://doi.org/10.1016/j.biopha.2023.115044.

[6]	A. Granic, C. Hurst, L. Dismore, R.M. Dodds, M.D. Witham, S.M. Robinson, A.A. Sayer, Advanced glycation end products in skeletal muscle health and sarcopenia: A systematic review of observational studies, Mech. Ageing Dev. 209 (2023) 111744. https://doi.org/10.1016/j.mad.2022.111744.

[7]	Bidirectional crosstalk between bone and muscle: the role of RANKL pathway in osteosarcopenia, J. Endocrinol. 262 (2024). https://doi.org/10.1530/JOE-24-0093.

[8]	V.V. Visconti, C. Greggi, I. Cariati, B. Gasperini, A. Mastrogregori, A. Botta, U. Tarantino, Deregulated Clusterin as a Marker of Bone Fragility: New Insights into the Pathophysiology of Osteoporosis, Genes 13 (2022) 652. https://doi.org/10.3390/genes13040652.

[9]	S. Pucci, C. Greggi, C. Polidoro, M.C. Piro, M. Celi, M. Feola, E. Gasbarra, R. Iundusi, F. Mastrangeli, G. Novelli, A. Orlandi, U. Tarantino, Clusterin silencing restores myoblasts viability and down modulates the inflammatory process in osteoporotic disease, J. Transl. Med. 17 (2019) 118. https://doi.org/10.1186/s12967-019-1868-5.

[10]	Association of Mesenchymal Stem Cell Communication with Key Genes COL1A1 and CD36 in Osteoporosis, Sciety (2025). https://sciety.org/articles/activity/10.21203/rs.3.rs-6227923/v1 (accessed July 19, 2025).

[11]	D. Liu, K. Wang, J. Wang, F. Cao, L. Tao, Identification of the molecular link: STAT3 is a shared key gene linking postmenopausal osteoporosis and sarcopenia, Bone Jt. Res. 13 (2024) 411–426. https://doi.org/10.1302/2046-3758.138.BJR-2023-0351.R2.

[12]	Y. Deng, Y. Wang, Q. Shi, Y. Jiang, Identification of hub genes associated with osteoporosis development by comprehensive bioinformatics analysis, Front. Genet. 14 (2023) 1028681. https://doi.org/10.3389/fgene.2023.1028681.

[13]	K. Polzer, L. Joosten, J. Gasser, J.H. Distler, G. Ruiz, W. Baum, K. Redlich, K. Bobacz, J.S. Smolen, W. van den Berg, G. Schett, J. Zwerina, Interleukin-1 is essential for systemic inflammatory bone loss, Ann. Rheum. Dis. 69 (2010) 284–290. https://doi.org/10.1136/ard.2008.104786.

[14]	Y. He, W. Duan, P. Xu, T. Lin, Q. Xiang, B. Dong, N. Ge, J. Yue, Exploring the impact of interleukins on sarcopenia development: A systematic review and meta-analysis, Exp. Gerontol. 193 (2024) 112480. https://doi.org/10.1016/j.exger.2024.112480.

[15]	B. Pineda, J.J. Tarín, C. Hermenegildo, P. Laporta, A. Cano, M.Á. García-Pérez, Gene-gene interaction between CD40 and CD40L reduces bone mineral density and increases osteoporosis risk in women, Osteoporos. Int. J. Establ. Result Coop. Eur. Found. Osteoporos. Natl. Osteoporos. Found. USA 22 (2011) 1451–1458. https://doi.org/10.1007/s00198-010-1324-0.

[16]	X. Xu, Y. Han, T. Zhu, F. Fan, X. Wang, Y. Liu, D. Luo, The role of SphK/S1P/S1PR signaling pathway in bone metabolism, Biomed. Pharmacother. Biomedecine Pharmacother. 169 (2023) 115838. https://doi.org/10.1016/j.biopha.2023.115838.

[17]	F. Pierucci, A. Frati, C. Battistini, F. Matteini, M.C. Iachini, A. Vestri, F. Penna, P. Costelli, E. Meacci, Involvement of released sphingosine 1-phosphate/sphingosine 1-phosphate receptor axis in skeletal muscle atrophy, Biochim. Biophys. Acta BBA - Mol. Basis Dis. 1864 (2018) 3598–3614. https://doi.org/10.1016/j.bbadis.2018.08.040.

[18]	TNF receptor-associated factors: promising targets of natural products for the treatment of osteoporosis - PMC, (n.d.). https://pmc.ncbi.nlm.nih.gov/articles/PMC12148923/ (accessed July 19, 2025).

[19]	M.N. Mansoori, P. Shukla, M. Kakaji, A.M. Tyagi, K. Srivastava, M. Shukla, M. Dixit, J. Kureel, S. Gupta, D. Singh, IL-18BP is decreased in osteoporotic women: Prevents Inflammasome mediated IL-18 activation and reduces Th17 differentiation, Sci. Rep. 6 (2016) 33680. https://doi.org/10.1038/srep33680.

[20]	T.-L. Ho, C.-H. Tang, S.L.-Y. Chang, C.-H. Tsai, H.-T. Chen, C.-M. Su, HMGB1 Promotes In Vitro and In Vivo Skeletal Muscle Atrophy through an IL-18-Dependent Mechanism, Cells 11 (2022) 3936. https://doi.org/10.3390/cells11233936.

[21]	T. Yu, X. You, H. Zhou, A. Kang, W. He, Z. Li, B. Li, J. Xia, H. Zhu, Y. Zhao, G. Yu, Y. Xiong, Y. Yang, p53 plays a central role in the development of osteoporosis, Aging 12 (2020) 10473–10487. https://doi.org/10.18632/aging.103271.

[22]	L. Di Renzo, S. Gratteri, F. Sarlo, A. Cabibbo, C. Colica, A. De Lorenzo, Individually tailored screening of susceptibility to sarcopenia using p53 codon 72 polymorphism, phenotypes, and conventional risk factors, Dis. Markers 2014 (2014) 743634. https://doi.org/10.1155/2014/743634.

[23]	W. Yu, M.-J. Tong, G.-H. Wu, T.-L. Ma, C.-D. Cai, L.-P. Wang, Y.-K. Zhang, J.-L. Gu, Z.-Q. Yan, FoxO3 Regulates Mouse Bone Mesenchymal Stem Cell Fate and Bone-Fat Balance During Skeletal Aging, Stem Cells Dev. 33 (2024) 365–375. https://doi.org/10.1089/scd.2024.0055.

[24]	B. Gellhaus, K.O. Böker, A.F. Schilling, D. Saul, Therapeutic Consequences of Targeting the IGF-1/PI3K/AKT/FOXO3 Axis in Sarcopenia: A Narrative Review, Cells 12 (2023) 2787. https://doi.org/10.3390/cells12242787.
