# Supplementary material for: Bridging bone and muscle: ontology-driven prioritization of candidate genes at the osteoporosis–sarcopenia interface by gene ontology reverse lookup
Source: Front Genet. 2026 Sep 25;17:1760184. doi: 10.3389/fgene.2026.1760184 (PMC13649218; doi:10.3389/fgene.2026.1760184)
Supplement: Supplementary file 2 [file Table3.docx]

**Supplementary material 3: KEGG over-representation analysis of the 37 prioritized candidate genes using ShinyGO version 0.82**

| Pathway | FE | *q* | nGenes | Pathway genes | Overlap genes |
| --- | --- | --- | --- | --- | --- |
| Malaria | 61.4 | 2.8×10⁻⁷ | 5 | 49 | *IL1B, IL18, MYD88, CD36, CD40LG* |
| NF-kappa B signalling pathway | 57.9 | 4.9×10⁻¹⁴ | 10 | 104 | *MALT1, IL1B, MYD88, PLCG2, PRKCQ, BTK, TRAF2, TNFSF11, RIPK1, TNFRSF11A* |
| Cytosolic DNA-sensing pathway | 47.8 | 7.6×10⁻⁷ | 5 | 63 | *PYCARD, IL1B, IL18, RIPK1, AIM2* |
| Legionellosis | 42.3 | 2.2×10⁻⁵ | 4 | 57 | *PYCARD, IL1B, IL18, MYD88* |
| NOD-like receptor signaling pathway | 39.5 | 7.7×10⁻¹⁵ | 12 | 183 | *NOD1, RBCK1, PYCARD, IL1B, IL18, MYD88, NOD2, CARD9, TRAF2, RIPK1, RIPK2, AIM2* |
| Inflammatory bowel disease | 37.1 | 3.1×10⁻⁵ | 4 | 65 | *IL1B, IL18, NOD2, STAT3* |
| Prolactin signaling pathway | 34.4 | 3.9×10⁻⁵ | 4 | 70 | *FOXO3, STAT3, TNFSF11, TNFRSF11A* |
| Shigellosis | 34.3 | 2.1×10⁻¹⁶ | 14 | 246 | *NOD1, RBCK1, MALT1, FOXO3, PYCARD, IL1B, IL18, MYD88, PLCG2, PRKCQ, TP53, TRAF2, RIPK1, RIPK2* |
| C-type lectin receptor signaling pathway | 28.9 | 8.7×10⁻⁶ | 5 | 104 | *MALT1, PYCARD, IL1B, PLCG2, CARD9* |
| Osteoclast differentiation | 28.7 | 7.6×10⁻⁷ | 6 | 126 | *IL1B, PLCG2, BTK, TRAF2, TNFSF11, TNFRSF11A* |
| Tuberculosis | 26.9 | 9.9×10⁻⁹ | 8 | 179 | *MALT1, IL1B, IL18, MYD88, NOD2, CARD9, RIPK2, SPHK1* |
| TNF signalling pathway | 26.9 | 1.2×10⁻⁵ | 5 | 112 | *IL1B, NOD2, TRAF2, RIPK1, IL18R1* |
| Epstein–Barr virus infection | 26.8 | 1.3×10⁻⁹ | 9 | 202 | *BCL2L11, MYD88, PLCG2, EIF2AK2, STAT3, BTK, TP53, TRAF2, RIPK1* |
| Necroptosis | 26.5 | 1.3×10⁻⁷ | 7 | 159 | *RBCK1, PYCARD, IL1B, EIF2AK2, STAT3, TRAF2, RIPK1* |
| Lipid and atherosclerosis | 25.3 | 1.7×10⁻⁹ | 9 | 214 | *PYCARD, IL1B, IL18, MYD88, STAT3, TP53, TRAF2, CD36, CD40LG* |

Table note: FE – fold enrichment; *q* – Benjamini-Hochberg adjusted *p*-value; nGenes - number of input genes overlapping the pathway; Pathway Genes - total genes annotated to that KEGG pathway; Overlap genes - overlapping input genes. Only terms meeting the pre-specified thresholds (*q* < 5×10⁻⁵ and fold-enrichment, FE > 25) are shown, ranked by FE. Pathways were identified with ShinyGO v0.82 using the KEGG Homo sapiens gene universe (hypergeometric enrichment test, default settings).
