# Supplementary material for: Bridging bone and muscle: ontology-driven prioritization of candidate genes at the osteoporosis–sarcopenia interface by gene ontology reverse lookup
Source: Front Genet. 2026 Sep 25;17:1760184. doi: 10.3389/fgene.2026.1760184 (PMC13649218; doi:10.3389/fgene.2026.1760184)
Supplement: Supplementary file 3 [file Table4.docx]

**Supplementary material 4. Summary of sarcopenia and osteoporosis-related GO-term assignments by mechanistic category and directionality**

**Table S1. Osteoporosis-related GO-term assignments by mechanistic category and directionality**

| **Category** | **Keywords** | **Stimulatory direction, n** | **Inhibitory direction, n** | **Total, n** |
| --- | --- | --- | --- | --- |
| Bone biology | Bone; remodelling | 5 | 17 | 22 |
| Bone-cell processes | Osteoblast; osteoclast; osteocyte | 9 | 7 | 16 |
| Mechanotransduction | Mechanical | 0 | 2 | 2 |
| Endocrine and hormonal regulation of bone | Estrogen (n=14); parathyroid hormone (n=13);  glucocorticoid (n=12);  vitamin D (n=4) | 23 | 20 | 43 |
| Inflammation and immune regulation | IL-1β; IL-6; TNF; M-CSF; regulatory T cell; Th17 | 10 | 7 | 17 |
| Gut microbiome-related processes | Short-chain fatty acid | 2 | 3 | 5 |
| Cellular senescence pathways | Senescence; apoptosis | 16 | 10 | 26 |
| Key signalling pathways | RANKL; Wnt; NF-κB; TGF-β; BMP; Notch | 15 | 13 | 28 |
| **Total** |  | 80 | 79 | 159 |

**Table note:** Stimulatory direction refers to GO biological process terms expected to promote or exacerbate the osteoporosis-related pathological state, whereas inhibitory direction refers to terms expected to oppose or reduce that state. Counts represent GO-term assignments in the original analysed input model; terms relevant to both states of interest are reported within the corresponding osteoporosis and sarcopenia assignments.

**Table S2. Sarcopenia-related GO-term assignments by mechanistic category and directionality**

| **Category** | **Keywords** | **Stimulatory direction, n** | **Inhibitory direction, n** | **Total, n** |
| --- | --- | --- | --- | --- |
| Muscle tissue processes | Muscle | 18 | 24 | 42 |
| Muscle-cell processes | Skeletal muscle cell; satellite cell; myoblast; myotube | 15 | 34 | 49 |
| Adipocyte-related processes | Adipocyte | 2 | 2 | 4 |
| Tendon-related processes | Tendon | 1 | 1 | 2 |
| Motor neuron / neuromuscular junction processes | Motor neuron; neuromuscular junction | 8 | 8 | 16 |
| Protein homeostasis | Ubiquitin; proteasome | 6 | 6 | 12 |
| Autophagy | Autophagy | 5 | 6 | 11 |
| Mitochondrial dysfunction and reactive oxygen species | Mitochondrion; reactive oxygen species; mitophagy | 18 | 19 | 37 |
| Endocrine and hormonal regulation | IGF-1 (n=2); androgen/testosterone (n=8); insulin signalling and secretion (n=8) | 11 | 7 | 18 |
| Key signalling pathways | PI3K; AMPK; Wnt; NF-κB; p38; Notch | 6 | 6 | 12 |
| Immune and cytokine-related processes | IL-15; macrophage | 2 | 2 | 4 |
| **Total** |  | 92 | 115 | 207 |

**Table note:** Stimulatory direction refers to GO biological process terms expected to promote or exacerbate the sarcopenia-related pathological state, whereas inhibitory direction refers to terms expected to oppose or reduce that state. Counts represent GO-term assignments in the original analysed input model; terms relevant to both states of interest are reported within the corresponding osteoporosis and sarcopenia assignments.
